# Preoperative Computed Tomography-Based Prediction and Patterns of Lymph Node Metastasis in Renal Pelvis and Ureteral Urothelial Carcinomas

**DOI:** 10.3390/cancers17071180

**Published:** 2025-03-31

**Authors:** Soojung Park, Deuk Jae Sung, Kyung Sook Yang, Yeo Eun Han, Ki Choon Sim, Na Yeon Han, Beom Jin Park, Min Ju Kim

**Affiliations:** 1 Department of Radiology, Anam Hospital, Korea University College of Medicine, 73 Goryeodae-ro, Seongbuk-gu, Seoul 02841, Republic of Korea; soojp124@gmail.com (S.P.); yeonny0714@korea.ac.kr (Y.E.H.); kspringsim@korea.ac.kr (K.C.S.); mammos@korea.ac.kr (N.Y.H.); bjp226@korea.ac.kr (B.J.P.); mjkim7@korea.ac.kr (M.J.K.); 2 Department of Biostatistics, Korea University College of Medicine, 73 Goryeodae-ro, Seongbuk-gu, Seoul 02841, Republic of Korea; myksyang@gmail.com

**Keywords:** CT, urothelial carcinoma, renal pelvis, ureter, metastasis, lymph node

## Abstract

**Background/Objectives:** The accurate preoperative prediction of lymph node (LN) metastasis is essential to optimizing surgical management in renal pelvis urothelial carcinoma (RPUC) and ureteral urothelial carcinoma (UUC). This study evaluates the predictive value of preoperative computed tomography (CT) findings in detecting LN metastasis and determining primary metastatic LN location based on the tumor site. **Methods:** This retrospective study included 48 RPUC and 97 UUC patients who underwent surgery with lymph node dissection (LND) between 2005 and 2023. Preoperative CT images were assessed for tumor size, location, multifocality, peritumoral fat infiltration, hydronephrosis grade, LN status, and metastatic LN location. Logistic regression and receiver operating characteristic (ROC) curve analyses identified predictive factors for LN metastasis, while Pearson’s chi-square and Fisher’s exact tests determined the association between locations of LN metastasis and primary tumor sites after categorizing UUC into upper and lower UUC. **Results:** In RPUC, 13 of 48 patients had LN metastasis, with tumor size and peritumoral fat infiltration emerging as significant predictors (*p* < 0.05). In UUC, 39 of 97 patients had LN metastasis, with tumor size and hydronephrosis grade being significant predictors (*p* < 0.001). An optimal tumor size threshold of 4 cm was identified for predicting LN metastasis in UUC, and 4.4 cm for RPUC. Additionally, a hydronephrosis grade of 3 or higher was found to be a strong predictor in UUC. ROC analysis showed high accuracy, yielding an AUC of 0.907 in RPUC and 0.904 in UUC. Cross-validation supported the robustness of these findings. Primary LN metastatic sites were predominantly ipsilateral hilar nodes in RPUC and ipsilateral pelvic nodes in lower UUC (*p* < 0.001). **Conclusions:** Preoperative CT imaging provides a reliable, noninvasive tool for predicting LN metastasis in RPUC and UUC. Identifying key imaging-based predictors can facilitate risk stratification and surgical decision-making, particularly regarding the necessity and extent of LND.

## Simple Summary

Lymph node (LN) metastasis is a critical prognostic factor in upper-tract urothelial carcinoma (UTUC), yet predicting its occurrence preoperatively remains challenging. This study evaluates the ability of computed tomography (CT) imaging to predict LN metastasis and determine metastatic LN locations in renal pelvis urothelial carcinoma (RPUC) and ureteral urothelial carcinoma (UUC). The findings reveal that tumor size (>4 cm) and hydronephrosis grade (≥3) significantly predict LN metastasis in UUC, while tumor size (>4.4 cm) and peritumoral fat infiltration are key predictors in RPUC. LN metastases predominantly occur in the ipsilateral hilar region for RPUC and the ipsilateral pelvic region for lower UUC. These results support a more tailored surgical approach for LN dissection, optimizing patient outcomes. By integrating imaging-based predictions, this study contributes to refining risk stratification and guiding clinical decision-making in UTUC management. Further prospective research is needed to validate these findings.

## 1. Introduction

Upper-tract urothelial carcinoma (UTUC) is a rare malignancy, accounting for approximately 5% of all urothelial cancers. [1]. Lymph node (LN) involvement significantly impacts survival outcomes in UTUC patients, making it a critical prognostic factor [2]. Despite the potential benefits of lymph node dissection (LND) in improving cancer outcomes, standardized guidelines for its application—including indications and extent—remain undefined in the management of UTUC [3,4]. Recent evidence suggests that template-based LND during nephroureterectomy may enhance LN staging and improve prognostic accuracy, particularly in high-risk UTUC cases [5]. Nevertheless, the therapeutic value of LND during nephroureterectomy continues to be widely debated [6,7,8]. The 2023 European Association of Urology guidelines endorse LND for high-risk, non-metastatic UTUC patients undergoing nephroureterectomy, although this recommendation is based on limited evidence [9].

The quest to pinpoint predictors of LN metastasis has underscored the significance of factors such as tumor stage, grade, presence of carcinoma in situ, lymphovascular invasion, and sessile tumor architecture [10,11,12]. Despite advancements in imaging technology, preoperative detection of LN metastases remains challenging. However, radiological features such as hydronephrosis have been associated with unfavorable pathological characteristics and can aid in preoperative risk stratification [13,14]. This underscores the potential of specific preoperative computed tomography (CT) findings to serve as predictors for LN involvement.

Renal pelvis urothelial carcinoma (RPUC) and ureteral urothelial carcinoma (UUC), while both are classified as UTUC, exhibit distinct clinical and pathological behaviors that influence their management and prognosis. LND is more commonly applied in cases of UUC, especially distal UUC, due to varied lymphatic drainage patterns and the ease of surgical access [15,16]. To date, no comprehensive studies have investigated the predictive value of CT findings for LN metastasis in distinct groups of UTUC patients categorized into RPUC and UUC. This study aimed to explore the association between preoperative CT variables and LN metastasis in these two distinct patient groups and to evaluate the primary location of LN metastasis according to primary tumor sites. By identifying reliable predictors on CT and primary locations of LN metastasis, we can help refine the necessity and extent of LND, thereby enhancing the overall management of UTUC patients.

## 2. Materials and Methods

### 2.1. Patient Selection

This retrospective, single-center study was approved by the institutional review board, which waived the requirement for informed consent.

We reviewed institutional patient records to identify all sequential cases of UTUC in individuals who had been diagnosed histopathologically between January 2005 and December 2023. Inclusion criteria were the following: (1) patients had undergone nephroureterectomy with LND and (2) availability of preoperative CT images obtained within one month prior to surgery. Initially, 171 patients were identified (RPUC, n = 61; UUC, n = 110). Exclusions were made for previous bladder urothelial carcinoma (RPUC, n = 5; UUC, n = 4), concurrent ureteral stones or percutaneous nephrostomy affecting hydronephrosis grading (RPUC, n = 2; UUC, n = 5), neoadjuvant chemotherapy (RPUC, n = 6; UUC, n = 3), and another coexistent tumor in pelvis (UUC, n = 1). Consequently, 48 RPUC and 97 UUC patients were included in the final analysis. The locations of metastatic LNs were recorded according to the pathological and surgical reports.

### 2.2. Imaging Acquisition

CT examinations were performed using multidetector CT scanners ranging from 16 to 128 channels (Somatom Sensation 16, Siemens Healthcare; Brilliance 64, Philips Medical Systems, Best, the Netherlands; and Somatom Definition Flash 128, Siemens Healthcare, Forchheim, Germany). The most frequently used scanning parameters were as follows: voltage, 120 kV; tube current, 300 mAs; section thickness, 5 mm; pitch and speed, 0.891:1; rotation time, 0.75 s; and collimation, 64 × 0.625 mm for 64-channel multidetector CT.

Patients underwent unenhanced CT initially, followed by contrast-enhanced CT using 300–350 mg/mL of iodine-based contrast agents (iohexol [Bonorex 300, Central Medical Service, Seoul, the Republic of Korea], iomeprol [Iomeron 300, Bracco Altana Pharma, Konstanz, Germany], or iobitridol [Xenetix 300, Guerbet, Villepinte, France]). The contrast medium was administered intravenously at a dose of 2 mL/kg and a rate of 3.0 mL/s. Corticomedullary and excretory phases were acquired 30–40 s and 300 s after injection, respectively.

### 2.3. Imaging Analysis

Two radiologists (D.J.S. with 23 years and S.P. with 4 years of experience in uroradiology) independently reviewed the CT images using a picture archiving and communication system (PACS) workstation. Any discrepancies were resolved by consensus. To address potential bias in the consensus process arising from the substantial difference in experience between the two readers, a third radiologist (K.C.S. with 14 years of experience in uroradiology) conducted an independent analysis of the CT images to assess additional interobserver agreement.

The assessed CT features included the presence of LN metastasis, tumor location, tumor size, presence of tumor multifocality, presence of peritumoral fat infiltration, hydronephrosis grade, and location of LN metastasis.

Lymphadenopathy on CT was determined when the short axis of the LN, averaged from measurements by the two radiologists, was 8 mm or greater. The location of lymphadenopathy was categorized into four types—hilar, upper retroperitoneal, pelvic, and others. We defined hilar LNs as those around the renal vessels; upper-retroperitoneal LNs as left paraaortic, aortocaval, retrocaval, and paracaval LNs; pelvis LNs as aortic bifurcation, iliac chain, and obturator LNs; and other LNs as cardiophrenic, retrocrural, gastrohepatic, mesenteric, and inguinal LNs. This simplified categorization was primarily based on the LN map proposed by Kondo et al. [11].

**Figure 1:**
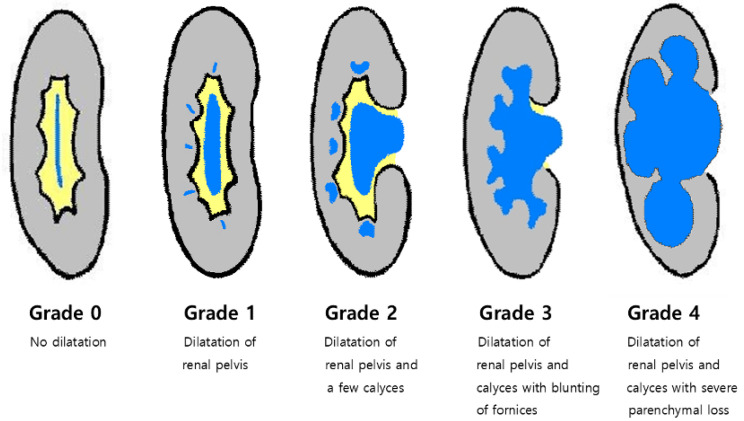
Schematic drawing of the Society for Fetal Urology hydronephrosis grading system.

Tumor size was defined as the largest length or width of the tumor, appearing as either a soft-tissue mass or wall thickening on axial, sagittal, or coronal CT images. This size was then averaged based on measurements by the two radiologists. The presence of tumor multifocality was confirmed by identifying multiple tumor sites within the upper urinary tract synchronously. For multifocal tumors, the size of the largest tumor was recorded, and its site was designated as the primary tumor location. Peritumoral fat infiltration was determined through imaging features such as irregular tumor margins, stranding or smudging of fat, loss of interface, or abnormal fat enhancement adjacent to the tumor. Hydronephrosis grade was assessed using the Society for Fetal Urology Hydronephrosis Grading System (Figure 1). For determining the primary location of LN metastasis according to tumor sites, UUC was categorized into upper and lower UUC based on the level of the sacroiliac joint because the ureter has a fairly long course from the upper retroperitoneum to the lower pelvis.

### 2.4. Statistical Analysis

Statistical analyses were performed using SAS version 9.4 (SAS Institute, Cary, NC, USA) and MedCalc version 18.5 (MedCalc Software Ltd., Belgium). A *p*-value of <0.05 was considered statistically significant. Continuous variables were checked for normal distribution using the Shapiro–Wilk test. Descriptive statistics such as mean, standard deviation, median, interquartile range, and frequencies with percentages were employed to summarize the data. Univariate logistic regression analysis was conducted to explore associations between imaging characteristics and LN metastasis. Variables with *p*-values below 0.1 were included in a multivariable logistic regression analysis, which was conducted using backward selection. A receiver operating characteristic (ROC) curve using the final model was constructed, and cross-validation was conducted by using leave-one-out cross-validation due to the limited sample size. A post hoc statistical power analysis was performed by using G*Power (version 3.1). Interobserver agreement was quantified using Cohen’s kappa statistics for nominal variables, including the presence of peritumoral fat infiltration, tumor multifocality, and hydronephrosis grade. Intraclass correlation was calculated for tumor size.

The scores were used to define agreement as follows: 0.41–0.60 denoted moderate agreement; 0.61–0.80, good agreement; and greater than 0.81, excellent agreement. Pearson’s chi-square test and Fisher’s exact test were used to analyze the association between the locations of the metastatic LN and primary tumor. Additionally, the diagnostic accuracy, sensitivity, and specificity of CT were calculated for detecting metastatic LN and organ-confined tumors (≤T2).

## 3. Results

### 3.1. Comparison of Clinical and Imaging Features

In 48 patients with RPUC, LN metastasis was pathologically confirmed in 13 patients after LND. The imaging and clinical features of these groups are shown in Table 1. Patients with LN metastasis exhibited significantly larger tumors and more frequent peritumoral fat infiltration compared to those without LN metastasis (*p* < 0.001).

For UUC, of the 97 assessed patients, 39 patients had pathologically confirmed LN metastasis following LND. Patients with LN metastasis demonstrated significantly larger tumors, higher hydronephrosis grades, more frequent peritumoral fat infiltration, and more frequent tumor multifocality, as detailed in Table 2 (all *p* < 0.001).

### 3.2. Logistic Regression Analyses

In patients with RPUC, univariate logistic regression analysis identified larger tumor size, peritumoral fat infiltration, and lymphadenopathy on CT as significantly associated with LN metastasis (all *p* < 0.01). Multivariable analysis revealed tumor size and tumor multifocality as independent predictors of LN metastasis. For UUC patients, the univariate analysis identified tumor size, peritumoral fat infiltration, hydronephrosis grade, tumor multifocality, and lymphadenopathy on CT as significant predictors of LN metastasis (all *p* < 0.01). Multivariable analysis confirmed tumor size and hydronephrosis grade as independent predictors. Detailed results of the logistic regression analyses are shown in Table 3.

### 3.3. Model Evaluation Using ROC Curve Analysis for LN Metastasis

**Figure 2:**
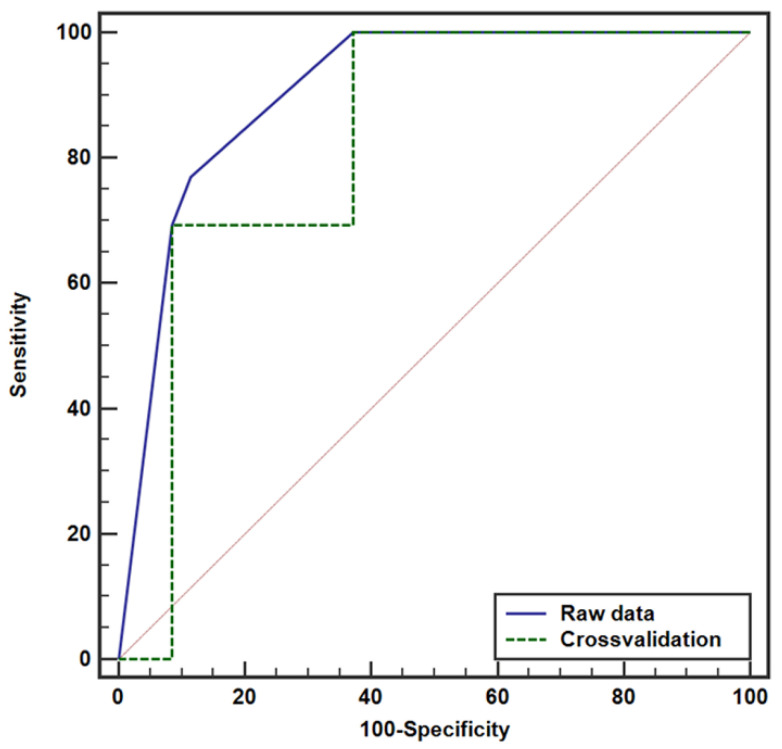
Receiver operating characteristic (ROC) curve for predicting lymph node metastasis in patients with renal pelvis urothelial carcinoma. The area under the curve (AUC) is 0.907 for raw data and 0.826 for cross-validation. The diagonal line indicates an AUC of 0.5, representing no diagnostic discrimination.

**Figure 3:**
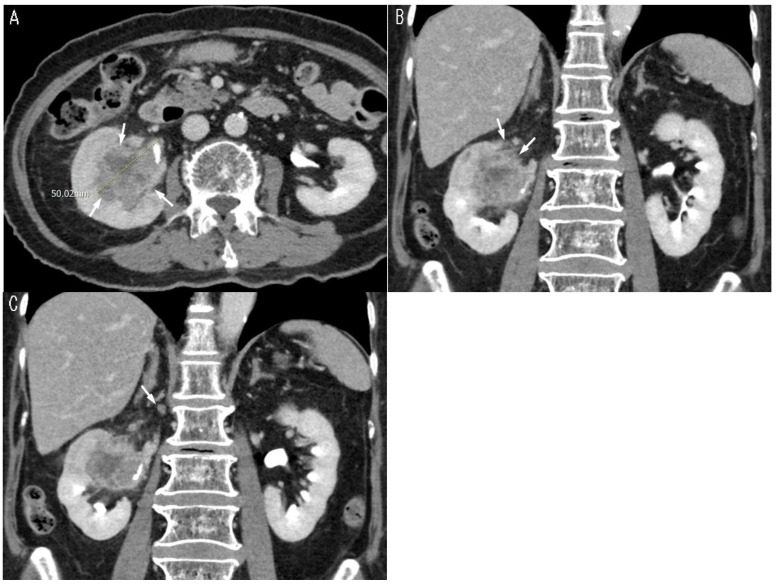
CT images of an 82-year-old female patient with renal pelvis urothelial carcinoma. (**A**) Axial preoperative CT image shows a partially ill-defined soft-tissue mass (arrows) in the right renal pelvis without pelvicalyceal dilatation. The mass measures 5 cm in maximum diameter and was confirmed as high-grade urothelial carcinoma. (**B**) Coronal CT image shows peritumoral fat infiltration (arrows) in the upper aspect of the soft tissue mass involving the right renal pelvis. (**C**) Coronal CT image shows a small right hilar lymph node (arrow), 4.5 mm in short-axis diameter, confirmed as metastatic following lymph node dissection.

Various combinations of variables were tested to identify the optimal model, as variables with the best cutoff values in univariate analysis do not always enhance performance in multivariable models. The final model was evaluated by incorporating tumor size with different cutoff values in each multivariable analysis. In patients with RPUC, ROC curve analysis revealed an area under the curve (AUC) of 0.907, setting the optimal cutoff of tumor size at 4.4 cm with the presence of peritumoral fat infiltration (Figure 2). Cross-validation maintained a robust AUC of 0.826. The AUC was 0.858 for tumor size by itself with the cutoff at 4.4 cm (Figure 3) (Table 4).

**Figure 4:**
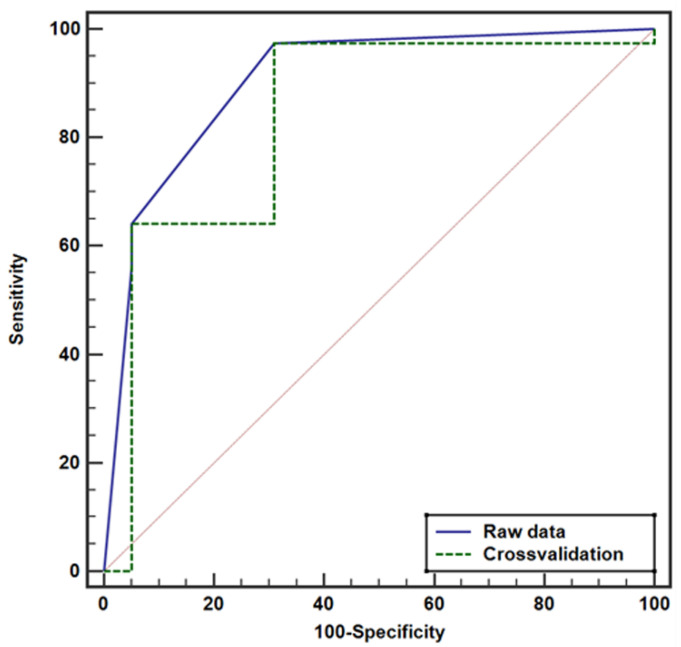
Receiver operating characteristic (ROC) curve for predicting lymph node metastasis in patients with ureteral urothelial carcinoma. The area under the curve (AUC) is 0.904 for raw data and 0.838 for cross-validation. The diagonal line indicates an AUC of 0.5, representing no diagnostic discrimination.

**Figure 5:**
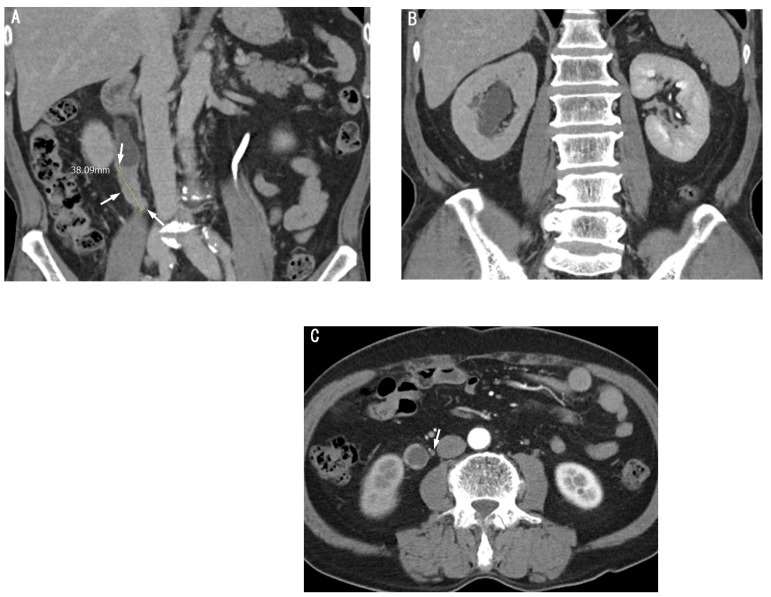
CT images of a 63-year-old male patient with ureteral urothelial carcinoma. (**A**) Coronal preoperative CT image shows a soft tissue mass (arrows) in the right upper ureter, causing upstream ureteral dilatation. The mass measured 3.8 cm and was pathologically confirmed as high-grade urothelial carcinoma. (**B**) Coronal CT image reveals marked dilatation of the renal pelvis and calyces with forniceal blunting, consistent with grade 3 hydronephrosis. (**C**) Axial CT image shows a small paracaval lymph node (arrow), 3 mm in short-axis diameter, confirmed as metastatic after lymph node dissection.

In patients with UUC, ROC analysis yielded an AUC of 0.904, setting an optimal cutoff of tumor size at 4 cm and a cutoff of hydronephrosis grade at 3 or higher (Figure 4). Cross-validation confirmed these results with an AUC of 0.838. The AUC was 0.832 for hydronephrosis grade by itself with the cutoff at grade 3 or higher (Figure 5) (Table 4). Detailed results of model evaluation are displayed in Table 5.

### 3.4. Interobserver Agreement Assessment

The two radiologists achieved good or excellent agreement for the CT variables in patients with RPUC, with kappa values of 0.819 for hydronephrosis grade, 0.792 for peritumoral fat infiltration, and 0.878 for tumor multifocality. For patients with UUC, there was excellent agreement on the CT variables, with kappa values of 0.864 for hydronephrosis grade, 0.856 for peritumoral fat infiltration, and 0.967 for tumor multifocality. Furthermore, the third radiologist also exhibited excellent agreement with the consensus data from the initial two radiologists for both RPUC and UUC. Detailed results are displayed in Table 6.

### 3.5. Post Hoc Statistical Power Analysis

Based on the distribution of tumor size categorized by the 4.4 cm cutoff and the presence of LN metastasis in patients with RPUC, the post hoc statistical power analysis yielded a value of 0.995. Similarly, in patients with UUC, the post hoc statistical power was calculated as 0.999 using a cutoff value of 4.0 cm.

### 3.6. Location of LN Metastasis

Pearson’s chi-square and Fisher’s exact tests showed that RPUC was associated with ipsilateral hilar metastatic LNs (*p* = 0.005) and lower UUC was associated with ipsilateral pelvic metastatic LNs (*p* < 0.001). There was no significant association between upper UUC and specific LN locations. Upper-retroperitoneal LNs and other locations LNs showed no significant association with the primary tumor sites, as detailed in Table 7.

### 3.7. CT Accuracy in LN Metastasis Detection and Local Staging

For diagnosing metastatic LN, CT had an accuracy of 80.8%, a sensitivity of 61.5%, and a specificity of 100% in patients with RPUC, and an accuracy of 83.3%, a sensitivity of 66.7%, and a specificity of 100% in patients with UUC. For distinguishing between organ-confined tumors (≤T2) and advanced tumors (≥T3), CT exhibited an accuracy of 80%, a sensitivity of 75%, and a specificity of 85% in patients with RPUC and an accuracy of 75.1%, a sensitivity of 80%, and a specificity of 70.2% in patients with UUC.

## 4. Discussion

Extensive research has explored LN metastasis and LND in UTUC, yet debates continue. LND is usually performed for high-risk UTUC, but, currently, there are no accepted guidelines on the indication or extent of LND. According to a systematic review, most studies reported the indication and extent to be at the “surgeon’s discretion”, based on the clinical stage, primary tumor location, and clinical information, such as comorbidities. The role of LND in UTUC is still controversial. A systematic review reported that LND has no therapeutic benefits, such as cancer-specific survival or overall survival, and that LND-related complications, such as numbness in the thigh, lymphorrhea, chyle leak, and retroperitoneal abscess, can occur [4]. In our institution, LND has not been routinely performed in patients with UTUC. In contrast, a growing number of studies have steadily explored the potential therapeutic benefits of LND in UTUC [17,18,19,20]. It is important to note that while these studies suggest potential therapeutic benefits of LND in UTUC, high-quality randomized controlled trials are still lacking. The AUA 2024 meeting discussions emphasized the ongoing uncertainty surrounding LND for UTUC due to the absence of robust randomized controlled data [21].

The European Association of Urology recommends template-based LND for high-risk, non-metastatic UTUC. High-risk factors include tumor stage, grade, size, location, multifocality, hydronephrosis, lymphovascular invasion, LN metastasis, and concomitant carcinoma in situ [9,13,14,22]. Many of these risk factors are assessable radiologically, leading our study to hypothesize that specific CT characteristics could predict LN metastasis, potentially expanding the pool of beneficiaries for LND by avoiding the pre-surgical misclassification of patients as low-risk.

RPUC and UUC, despite sharing a common origin, display varied clinical behaviors, prognoses, and risks of LN metastasis. UUC is generally associated with a poorer prognosis and a higher incidence of multifocality than RPUC [23,24,25]. Anatomical differences in the upper urinary tract also contribute to varying risks of tumor invasion and metastasis. This heterogeneity necessitates a specialized approach to LND that considers tumor location, highlighting the importance of our study’s division of UTUC into RPUC and UUC groups for analysis.

Accurately staging UTUC preoperatively is crucial for deciding LND candidacy. However, current imaging modalities fall short of detecting microscopic invasions and differentiating between inflammatory changes and peritumoral fat infiltration, which can lead to overstaging [14,24]. In this study, the accuracy of CT in diagnosing localized and organ-confined UTUC (≤T2) was less than 80%. This challenge is underscored by findings that a significant portion of tumors initially staged as cT1 or lower later upstaged following surgery, thus increasing their likelihood of regional metastasis [26].

Tumor size is a well-established prognostic factor in UTUC. Several studies have reported that tumor sizes between 3 and 3.5 cm are associated with poor outcomes, including extraurothelial recurrence, with many identifying 3 cm as a key cutoff value [27,28,29]. However, these studies did not specifically focus on tumor size as a predictor of lymph node metastasis, and, in some cases, the 3 cm or 3.5 cm thresholds reflected median tumor sizes rather than optimal predictive values. More recently, one study identified a tumor size cutoff of 5 cm as optimal for predicting adverse outcomes in UTUC, based on univariate, multivariate, and pairwise logistic regression analyses [30]. In contrast, the optimal cutoff values in our study were slightly lower, at 4.4 cm for RPUC and 4 cm for UUC, respectively. In UTUC, tumor dimensions have been linked to more aggressive biological characteristics, with greater sizes being more frequently associated with lymphovascular invasion, which is considered a potential precursor to lymph node metastasis [27].

Hydronephrosis is associated with advanced disease and poor outcomes in UTUC, even though underlying mechanisms remain unclear. It is hypothesized that HN could lead to outward distension and progressive thinning of the inherently narrow ureter or renal pelvis walls, potentially promoting the dissemination of malignant cells to nearby or distant sites. Additionally, the elevated centrifugal pressure resulting from HN may induce retrograde flow within lymphatic and vascular channels, thereby enhancing the likelihood of tumor cell spread [13]. A recent meta-analysis confirmed its importance as a predictor of LN metastasis and advanced stage [14]. Our study found that hydronephrosis grade was a significant predictor of LN metastasis in patients with UUC, particularly for grades ≥3, but not in patients with RPUC. This distinction is logical, as only RPUC that invades the ureteropelvic junction can lead to hydronephrosis. In contrast, UUC is more strongly associated with hydronephrosis, as even relatively small tumors can obstruct the ureter’s narrow lumen, causing upstream dilation.

The size of LN on CT scans is an important criterion for assessing the likelihood of metastasis and can also influence the decision to perform LND. Generally, LN metastasis in urothelial carcinoma is diagnosed using CT, with a size cutoff of 8 mm in short-axis diameter [31]. However, CT has limitations in accurately detecting LN metastases in UTUC. While the presence of lymphadenopathy on CT strongly predicts metastases, smaller metastatic nodes may remain undetected [32,33]. In our study, while CT lymphadenopathy was a strong predictor of LN metastasis in univariate analyses, it did not remain significant in multivariable models. This may stem from CT’s limited sensitivity (61.5% for RPUC, 66.7% for UUC) using an 8 mm size cutoff in detecting metastatic LN. Conversely, enlarged lymph nodes on CT are not always malignant, as a substantial number of clinically suspected LN metastases represent false positives due to reactive or inflammatory lymphadenopathy [21,34]. Moreover, tumor size, peritumoral fat infiltration, and hydronephrosis grade—variables that retained significance in our multivariable analyses—likely capture more direct aspects of tumor aggressiveness and lymphatic spread, overshadowing the contribution of LN enlargement. This finding underscores the importance of a comprehensive CT-based evaluation of tumor-specific features to enhance preoperative risk stratification and inform decisions regarding LND. To address the limitations of LN size as a predictor, future studies could explore advanced imaging techniques, such as positron emission tomography with radiotracers like 18F-FDG, which have shown promise in detecting micrometastases in patients with urothelial carcinoma [35].

Peritumoral fat infiltration identified on CT is a notable radiological indicator linked to the pathologic T3 stage in UTUC, indicating tumor extension into the peripelvic or periureteral adipose tissue. In correlating CT findings with pathology, one study reported a sensitivity and specificity of 87.5% and 92.9%, respectively, for identifying tumors at or above stage pT3 in UTUC [36]. In contrast, our study demonstrated lower sensitivity and specificity, with results of 75% and 85% for RPUC, and 80% and 70.2% for UUC. Although the relationship between peritumoral fat infiltration observed on CT and LN metastasis is not well established for RPUC and UUC, invasion into peripelvic or periureteral fat is linked to an increased risk of metastasis in UTUC [37,38]. Furthermore, LN involvement is more frequently observed in higher T stages, such as T3 and T4, among patients with UTUC [39]. Our study found a significant association between peritumoral fat infiltration and LN metastasis in patients with RPUC.

The latest AUA/SUO guidelines for non-metastatic UTUC classify the disease into low- and high-risk categories based on tumor biopsy grade. These risk groups are further sub-stratified based on prognosis—favorable or unfavorable—according to factors such as urinary obstruction, tumor multifocality, contralateral UTUC, and involvement of the urinary bladder [40]. LND is strongly recommended for all high-risk UTUC cases, regardless of the sub-stratification, thereby limiting the role of radiological evaluation in selecting candidates for LND. In defining high-risk UTUC, to which template LND is applicable, the most recent EAU guidelines consider tumor grade, assessed through cytology or biopsy, as the primary surrogate marker for identifying such cases. However, the level of evidence to individually consider tumor size, multifocality, and hydronephrosis as a surrogate for high-risk progression remains low. Therefore, in the presence of low-grade disease associated with these factors, a shared decision-making process with the patient is important to agree on the therapeutic strategy [41].

The pattern of LN metastasis in UTUC plays a critical role in guiding template-based LND, which is essential for accurate staging and potential therapeutic benefits. One study reported that in left-sided RPUC, 50% of all metastatic LNs were identified in the ipsilateral hilar region, whereas in right-sided RPUC, 44.1% of metastatic LNs were located in the paracaval region, and 22.1% were identified in the right hilar region [33]. In this study, LN metastasis was significantly more common in the ipsilateral hilar region among patients with RPUC, while in patients with lower UUC, the primary and most significant site of LN metastasis was the ipsilateral pelvic region, particularly in the iliac chains and obturator area. These findings are consistent with previous review articles on the anatomical templates for LND in UTUC [42,43]. Upper UUC has been reported to metastasize to various regions, including the hilar and para-aortic nodes, but without a consistent pattern [33]. The lack of a statistically significant correlation between upper UUC and specific LN locations in our study aligns with the existing literature.

This study has several limitations. First, its retrospective design inherently introduces selection bias, particularly because not all UTUC patients at our institution routinely underwent LND following nephroureterectomy. The omission of LND in UTUC patients is a common practice worldwide, reflecting the ongoing debate regarding its clinical utility. Second, discrepancies in CT findings, such as hydronephrosis grade, peritumoral fat infiltration, and tumor multifocality, were resolved through consensus review. While the two reviewers demonstrated good to excellent interobserver agreement for CT variables, the consensus approach may have introduced bias by potentially masking the true level of diagnostic performance—especially if interpretations were disproportionately influenced by more experienced radiologists. Therefore, to address this concern, a third reviewer independently assessed the CT findings, showing excellent agreement with the initial two reviewers. Finally, CT has inherent limitations in accurately measuring primary tumor size, particularly in the ureter, due to its non-perpendicular course. This anatomical challenge may lead to an underestimation of the actual tumor size on imaging. Despite these limitations, our study provides valuable insights into the imaging-based prediction and patterns of LN metastasis in RPUC and UUC, emphasizing the need for further research to refine diagnostic and therapeutic strategies

## 5. Conclusions

This study identifies significant predictors of LN metastasis in patients with RPUC and UUC based on preoperative CT findings. For RPUC, a tumor size > 4.4 cm and peritumoral fat infiltration were significant indicators, while for UUC, a tumor size > 4 cm and a hydronephrosis grade ≥ 3 were strong predictive factors of LN metastasis. Our findings also reveal distinct patterns of LN metastasis: RPUC primarily metastasizes to ipsilateral hilar LNs, whereas lower UUC predominantly spreads to ipsilateral pelvic LNs. These insights can enhance risk stratification and guide surgical decision-making, particularly regarding the necessity and extent of LND. Moreover, integrating imaging-based predictions into clinical practice has the potential to refine LND guidelines for RPUC and UUC, allowing for a more tailored approach based on tumor location, rather than a uniform, one-size-fits-all strategy. Larger prospective, multicenter studies are necessary to validate these findings.

## Figures and Tables

**Table 1:** Comparisons of clinical characteristics and CT findings between the patients with and without LN metastasis in RPUC.

	LN Metastasis (−) (N = 35)	LN Metastasis (+) (N = 13)	Total (N = 48)	*p*-Value
Sex				0.739 ^1^
Male	29 (72.5%)	11 (78.6%)	40 (74.1%)	
Female	11 (27.5%)	3 (21.4%)	14 (25.9%)	
Age (years)	66.5 ± 11.07	64.4 ± 15.55	65.9 ± 12.27	0.579 ^4^
Tumor size (cm)	3.2 (1.49)	5.2 (1.93)	3.7 (1.81)	<0.001 ^3^
LN size in short axis				<0.001 ^1^
<8 mm	40 (100.0%)	6 (42.9%)	46 (85.2%)	
≥8 mm	0 (0.0%)	8 (57.1%)	8 (14.8%)	
Hydronephrosis grade (0~4)	0.6 (1.28)	1.0 (1.66)	0.7 (1.38)	0.439 ^3^
Hydronephrosis grade				0.429 ^1^
0	32 (80.0%)	10 (71.4%)	42 (77.8%)	
1	0 (0%)	0 (0%)	0 (0%)	
2	3 (7.5%)	0 (0.0%)	3 (5.6%)	
3	2 (5.0%)	2 (14.3%)	4 (7.4%)	
4	3 (7.5%)	2 (14.3%)	5 (9.3%)	
Peritumoral fat infiltration				0.002 ^2^
(−)	25 (62.5%)	2 (14.3%)	27 (50.0%)	
(+)	15 (37.5%)	12 (85.7%)	27 (50.0%)	
Tumor multiplicity				0.173 ^1^
1	37 (92.5%)	11 (78.6%)	48 (88.9%)	
≥2	3 (7.5%)	3 (21.4%)	6 (11.1%)	

LN = lymph node, RPUC= renal pelvis urothelial carcinoma. Data are mean ± standard deviation (SD) for age, median (interquartile range) for tumor size, hydronephrosis grade, and number of patients (%) for categorical variables. ^1^ Chi-square *p*-value; ^2^ Fisher exact *p*-value; ^3^ Wilcoxon rank sum *p*-value; ^4^ equal variance two-sample *t*-test.

**Table 2:** Comparisons of clinical characteristics and CT findings between the patients with and without LN metastasis in UUC.

	LN Metastasis (−) (N = 58)	LN Metastasis (+) (N = 39)	Total (N = 97)	*p*-Value
Sex				0.161 ^1^
Male	38 (65.5%)	20 (51.3%)	58 (59.8%)	
Female	20 (34.5%)	19 (48.7%)	39 (40.2%)	
Age (years)	70.0 ± 10.28	68.8 ± 10.11	69.5 ± 10.17	0.559 ^4^
Tumor size (cm)	1.9 (1.3, 2.4)	5.4 (3.3, 7.0)	2.4 (1.7, 4.8)	<0.001 ^3^
LN size in short axis				<0.001 ^1^
<8 mm	58 (100.0%)	13 (33.3%)	71 (73.2%)	
≥8 mm	0 (0.0%)	26 (66.7%)	26 (26.8%)	
Hydronephrosis grade (0~4)	2.0 (1.0, 3.0)	4.0 (3.0, 4.0)	3.0 (2.0, 4.0)	<0.001 ^3^
Hydronephrosis grade				<0.001 ^1^
0	8 (13.8%)	1 (2.6%)	9 (9.3%)	
1	10 (17.2%)	0 (0.0%)	10 (10.3%)	
2	22 (38.0%)	3 (7.7%)	25 (25.8%)	
3	10 (17.2%)	11 (28.2%)	21 (21.6%)	
4	8 (13.8%)	24 (61.5%)	32 (33.0%)	
Peritumoral fat infiltration				<0.001 ^2^
(−)	35 (60.3%)	8 (20.5%)	43 (44.3%)	
(+)	23 (39.7%)	31 (79.5%)	54 (55.7%)	
Tumor multiplicity				<0.001 ^1^
1	53 (91.4%)	25 (64.1%)	78 (80.4%)	
≥2	5 (8.6%)	14 (35.9%)	19 (19.6%)	

LN = lymph node, UUC = ureteral urothelial carcinoma. Data are mean ± standard deviation (SD) for age, median (interquartile range) for tumor size, hydronephrosis grade, and number of patients (%) for categorical variables. ^1^ Chi-square *p*-value; ^2^ Fisher exact *p*-value; ^3^ Wilcoxon rank sum *p*-value; ^4^ equal variance two-sample *t*-test.

**Table 3:** Univariate and multivariable logistic regression analyses of CT findings for LN metastasis in RPUC and UUC.

	Univariate Analysis		Multivariable Analysis	
Variable	OR (95% CI)	*p*-Value	OR (95% CI)	*p*-Value
In RPUC				
Tumor multifocality	4.95 (0.72, 33.90)	0.103		
Tumor size (>4.4 cm)	25.83 (4.92, 135.59)	<0.001	14.42 (2.41, 86.10)	0.003
Peritumoral fat infiltration	23.00 (2.66, 198.66)	0.004	11.35 (1.10, 116.83)	0.041
Hydronephrosis grade (0~4)	1.47 (0.92, 2.35)	0.112		
Lymphadenopathy	109.74 (4.66, >999.99)	0.004		
In UUC				
Tumor multifocality	5.93 (1.92, 18.3)	0.002		
Tumor size (>4 cm)	32.74 (8.63, 124.22)	<0.0001	19.93 (4.65, 85.55)	<0.001
Peritumoral fat infiltration	5.90 (2.31, 15.08)	<0.001		
Hydronephrosis grade (≥3)	19.44 (6.01, 62.94)	<0.0001	11.69 (3.09, 44.15)	<0.001
Lymphadenopathy	229.8 (12.47, >999.99)	<0.001		

OR = odds ratio, CI = confidence interval, LN = lymph node, RPUC = renal pelvis urothelial carcinoma, UUC = ureteral urothelial carcinoma.

**Table 4:** Optimal cutoff value and corresponding sensitivity, specificity, PPV, and NPV of each CT variable for LN metastasis in univariate analysis.

Variable	AUC (95% CI)	*p*-Value	Criterion	Sensitivity (95% CI)	Specificity (95% CI)	PPV (95% CI)	NPV (95% CI)
In RPUC							
Tumor size	0.858	<0.0001	>4.4	76.9	88.6	71.4	91.2
	(0.727, 0.942)			(46.2, 95.0)	(73.3, 96.8)	(48.7, 86.8)	(79.2, 96.6)
Hydronephrosis grade	0.593	0.219	≥3	30.8	91.4	57.1	78.0
	(0.442, 0.733)			(9.1, 61.4)	(76.9, 98.2)	(25.6, 83.8)	(70.9, 83.8)
Tumor multifocality	0.587	0.175	≥1	23.1	94.3	60.0	76.7
	(0.436, 0.727)			(5.0, 53.8)	(80.8, 99.3)	(22.0, 88.9)	(70.8, 81.8)
Lymphadenopathy	0.808	<0.001	>0	61.5	100	100	87.5
	(0.668, 0.907)			(31.6, 86.1)	(90.0, 100)	(63.1, 100)	(77.9, 93.3)
In UUC							
Tumor size	0.904	<0.0001	>3	79.5	93.1	88.6	87.1
	(0.828, 0.955)			(63.5, 90.7)	(83.3, 98.1)	(74.8, 95.3)	(78.4, 92.6)
Hydronephrosis grade	0.832	<0.0001	≥3	89.7	69.0	66.0	90.9
	(0.743, 0.900)			(75.8, 97.1)	(55.5, 80.5)	(56.6, 74.3)	(79.5, 96.3)
Tumor multifocality	0.636	0.0016	≥1	35.9	91.4	73.7	67.9
	(0.533, 0.732)			(21.2, 52.8)	(81.0, 97.1)	(52.3, 87.7)	(62.3, 73.1)
Lymphadenopathy	0.833	<0.0001	>0	66.7	100	100	81.7
	(0.744, 0.901)			(49.8, 80.9)	(93.8, 100)	(86.8,100)	(74.1, 87.4)

PPV = positive predictive values, NPV = negative predictive values, CI = confidence interval, LN = lymph node, RPUC = renal pelvis urothelial carcinoma, UUC = ureteral urothelial carcinoma.

**Table 5:** Performance of the final model of CT variables for predicting LN metastasis in patients with RPUC.

	Raw Data	Cross-Validation
In RPUC (Tumor Size > 4.4 cm, Peritumoral Fat Infiltration)		
AUC (95% CI)	0.907 (0.787, 0.971)	0.826 (0.690, 0.920)
*p*-value	<0.0001	<0.0001
Criterion	≥0.2084	≥0.0233
Sensitivity (95% CI)	76.9 (46.2, 95.0)	100 (75.3, 100.0)
Specificity (95% CI)	88.6 (73.3, 96.8)	62.9 (44.9, 78.5)
PPV (95% CI)	71.4 (48.7, 86.8)	50.0 (39.4, 60.6)
NPV (95% CI)	91.2 (79.2, 96.6)	100 (84.6, 100.0)
In UUC (Tumor Size > 4 cm, Hydronephrosis Grade ≥ 3)		
AUC (95% CI)	0.904 (0.828, 0.955)	0.838 (0.749, 0.905)
*p*-value	<0.0001	<0.0001
Criterion	≥0.058	≥0.059
Sensitivity (95% CI)	97.4 (86.5, 99.9)	97.4 (86.5, 99.9)
Specificity (95% CI)	69.0 (55.5, 80.5)	69.0 (55.5, 80.5)
PPV (95% CI)	67.9 (58.9, 75.7)	67.9 (58.9, 75.7)
NPV (95% CI)	97.6 (85.2, 99.6)	97.6 (85.2, 99.6)

AUC = area under the curve, PPV = positive predictive values, NPV = negative predictive values, CI = confidence interval, LN = lymph node, RPUC = renal pelvis urothelial carcinoma, UUC = ureteral urothelial carcinoma.

**Table 6:** Agreement analysis between the consensus data from two radiologists and the third radiologist’s data.

Data	Variable	Coefficient (95% CI)	*p*-Value
RPUC	Hydronephrosis grade	0.940 (0.822, 1.000) ^a^	<0.0001
	Peritumoral fat infiltration	0.917 (0.804, 1.000) ^a^	<0.0001
	Multifocality	0.813 (0.558, 1.000) ^a^	<0.0001
	Tumor size	0.999 (0.9989, 0.9996) ^b^	<0.0001
UUC	Hydronephrosis grade	0.959 (0.914, 1.000) ^a^	<0.0001
	Peritumoral fat infiltration	0.938 (0.868, 1.000) ^a^	<0.0001
	Multifocality	0.937 (0.851, 1.000) ^a^	<0.0001
	Tumor size	0.999 (0.9987, 0.9996) ^b^	<0.0001

^a^ Kappa (95% CI); ^b^ intraclass correlation coefficient (95% CI).

**Table 7:** Cross-tabulation of metastatic lymph node location and primary tumor site in patients with upper-tract urothelial carcinoma (n = 145 patients).

Primary Tumor	Ipsilateral Hilar LN		*p*-Value	Contralateral Hilar LN		*p*-Value
	(−)	(+)		(−)	(+)	
Lower UCC	75 (57.7%)	1 (6.7%)	<0.0001	75 (52.4%)	1 (50.0%)	1.000
Upper UUC	20 (15.4%)	1 (6.7%)		21 (14.7%)	0 (0.0%)	
RPUC	35 (26.9%)	13 (66.7%)		47 (32.9%)	1 (50.07%)	
RPUC vs. Others			<0.0001			1.000
Upper UUC vs. Others			0.697			1.000
Lower UUC vs. Others			<0.0001			1.000
**Primary Tumor**	**Ipsilateral Upper-Retroperitoneal LN**		***p*** **-Value**	**Contralateral Upper-Retroperitoneal LN**		***p*** **-Value**
	**(−)**	**(+)**		**(−)**	**(+)**	
Lower UCC	66 (52.4%)	10 (52.6%)	0.215	74 (52.5%)	2 (50.0%)	0.627
Upper UUC	16 (12.7%)	5 (26.3%)		20 (14.2%)	1 (25.0%)	
RPUC	44 (34.9%)	4 (21.1%)		47 (33.3%)	1 (25.0%)	
RPUC vs. Others			0.231			1.000
Upper UUC vs. Others			0.155			0.469
Lower UUC vs. Others			1.000			1.000
**Primary Tumor**	**Ipsilateral Pelvic LN**		***p*** **-Value**	**Contralateral Pelvic LN**		***p*** **-Value**
	**(−** **)**	**(+)**		**(−)**	**(+)**	
Lower UCC	46 (40.4%)	30 (96.8%)	<0.0001	76 (52.4%)	0 (0.0%)	N/A
Upper UUC	20 (17.5%)	1 (3.2%)		21 (14.5%)	0 (0.0%)	
RPUC	48 (42.1%)	0 (0.0%)		48 (33.1%)	0 (0.0%)	
RPUC vs. Others			<0.0001			N/A
Upper UUC vs. Others			0.047			N/A
Lower UUC vs. Others			<0.0001			N/A

Fisher’s exact test was used if any of the cells had a frequency of less than 5. Pearson’s chi-square test was used in other groups.

## Data Availability

Data can be available on reasonable request to the corresponding author.
